# Depressive symptoms and their sociodemographic determinants among people living with HIV/AIDS in Bangladesh: a cross-sectional study

**DOI:** 10.12688/f1000research.108557.3

**Published:** 2023-04-17

**Authors:** Rokshana Rabeya, Nur Alam, Zannatul Ferdous Sonia, Dipa Rani Mohajon, Yasin Arafat, Md. Kamrul Hasan, Mohammad Delwer Hossain Hawlader

**Affiliations:** 1 Department of Public Health Nutrition, Primeasia University, Dhaka, 1213, Bangladesh; 2 Department of Public Health, North South University, Dhaka, 1229, Bangladesh

**Keywords:** HIV/AIDS, depression, depressive symptoms, acquired immune deficiency syndrome (AIDS), Bangladesh

## Abstract

**Background**: HIV is a chronic disease with a profound social impact due to its strong ties with sexual and societal stigmatized behavior, such as illegal drug use and sexual promiscuity. Depression is one of the major disabling factors in chronic illnesses. Depression and anxiety disorders are more common among people living with HIV than the non-infected individuals. This study aimed to determine the prevalence of depression and its associated factors among people living with HIV/AIDS in Bangladesh.

**Methods**: This cross-sectional study, which took place in Dhaka, Bangladesh, from July to December 2020, included 338 HIV-positive people. The method used was a simple random sampling technique. The Beck Depression Inventory assessed depression in HIV-positive people (BDI).

**Results**: More than 62 percent of the 338 people surveyed had severe depression, 30.5 percent had moderate depression, 5.6 percent had mild depression, and 1.8 percent had no depression. Age, being a male, being married, and having a low monthly income were all significant predictors of depression.

**Conclusions**: This study found that depressive symptoms are highly prevalent among HIV-positive patients in Bangladesh. The authors recommend that health care providers address depressive disorders for people with HIV/ AIDS comprehensively.

## Introduction

Infection with the human immunodeficiency virus (HIV) causes acquired immune deficiency syndrome (AIDS) ( Krämer, Kretzschmar, & Krickeberg, 2010). Around 38 million people worldwide are infected with HIV ( Global Aids Update 2020, 2020). HIV is transmitted primarily through unprotected sex, contaminated blood transfusions, hypodermic needles, and mother-to-child transmission during pregnancy, delivery, or breastfeeding ( Rom & Markowitz, 2007). AIDS was first recognized as a new disease in 1981, when an increasing number of young homosexual men died of unusual opportunistic infections and rare cancers ( Sharp & Hahn, 2011). According to the World Health Organization (WHO), 36.7 million people worldwide live with HIV and AIDS, with 1.1 million dying in 2015 ( World Health Organization, 2018). In Bangladesh, the first case of HIV was discovered in 1989 ( Goldberg, 2010).

Depression (also called major depressive disorder or clinical depression) is a common but serious mood disorder. It causes severe symptoms that affect how you feel, think, and handle daily activities, such as sleeping, eating, or working ( https://www.nimh.nih.gov/health/topics/depression). It also has an impact on social behavior and physical well-being. It affects people of all ages, including children and teenagers ( Deshmukh, Borkar, & Deshmukh, 2017). Depression is common among HIV-positive people ( Deshmukh, Borkar, & Deshmukh, 2017). Depressed mood, loss of interest or pleasure, decreased energy, guilt or low self-worth, disturbed sleep or appetite, and poor concentration is all symptoms ( World Health Organization, 2017). It obstructs daily life and lowers life quality. People living with HIV (PLHIV) had more mental health problems than people not infected with the virus, with those who had fewer problems were less likely to be poor and more likely to be employed, educated, and on antiretroviral therapy (ART). Psychiatric morbidity is linked with several factors: being female, being in poor health, receiving poor-quality health care, and lacking material and emotional support from family and friends ( Brandt, 2009).

In PLWHA, depression is linked to increased morbidity and mortality, as well as poor adherence to antiretroviral therapy (ART), quality of life (QoL), and health-related quality of life (AQoL) ( Abas, Ali, Nakimuli-Mpungu, & Chibanda, 2014). The financial cost of HIV treatment for the victim/patient is enormous, frequently leading to poverty for the sufferer and his or her family. PLWHA means People living with HIV/AIDS ( Dahlui *et al.*, 2015). Married people are more likely to have stigma on PLWHA and are more likely to blame PLWHA for bringing the disease to the community. Also about half of the population discriminates against PLWHA ( Dahlui *et al.*, 2015). Even though depression among HIV patients is widespread in various countries, there is little evidence from Bangladesh. As a result, we conducted this research to fill a gap that may provide evidence for future effective HIV/AIDS prevention and treatment.

## Methods

From July to December 2020, an institution-based cross-sectional study among PLWHA in Bangladesh was conducted. Considering 67.3% population prevalence ( Rai & Verma, 2015), 5% error, and 95% confidence interval, our sample size was 338. We conducted this study in all drop-in centers (DIC) of CARE Bangladesh located in Chankharpul, Swamibag, Dholpur, Hazaribagh, Noya Bajar, and Tongi of Dhaka city. We recruited adult males, females, and transgender people who were advised for routine checkups in those centers. HIV-positive patients who were not willing to participate in this study were excluded.

For this study, a purposive sampling technique was applied for selecting the HIV working organization, and after that, a simple random sampling technique was applied to recruit the study participants. A written, structured questionnaire based on the objectives and variables was used for data collection (see extended data). Only close-ended questionnaires were used to assemble data, and the interview was completed in the local language. Questionnaires were first prepared in English and then translated into the local language Bangla and again back-translated into English to see the accuracy of Bangla translation.

The Statistical Package for Social Science (SPPS) version 25 was used to compile and analyze the data for this study. The questionnaire and data are available online ( Rabeya *et al.*, 2021, 2022). A chi-square test or Fisher exact determined the relationship between categorical variables. The presence and strength of association between independent variables and the severe depression category were determined using crude and adjusted odds ratios with a 95 percent confidence interval (CI). Variables with a “p-value” of less than 0.05 were considered significant in the bivariate logistic model.

### Ethics approval and consent to participate

The Institutional Review Board (IRB) of Primeasia University, Dhaka, Bangladesh, approved this study. The reference number is PAU/IEAC/22/103. Prior to data collection, we received approval from CARE Bangladesh addition to this approval. CARE Bangladesh is a humanitarian organization to improve the socioeconomic status of women and the marginalized population in Bangladesh.

Additionally, each participant was aware of the aim of the study, as well as they signed in the written informed consent form prior to providing information.

## Results

Table 1 shows that a total of 338 male, female, and transgender HIV-positive respondents aged between 18 to more than 50 years were enrolled in the study. Demographic characteristics of the subject (n=338) in this cross-sectional study show that most participants (35.8%) belonged to age groups of 18 to 30 years, 31 to 40 years 35.8%, 41 to 50 20.4%, and 50 and above were 8.0%. The mean age of the participants was 35.6 (±9.9) years. The study revealed that 297 (87.95%) were male, whereas 20 (5.95%) were female, and 21 (6.2%) were transgender. Among 338 participants, 116 (34.3%) were illiterate, 173 (51.2%) were educated up to secondary school level (10 ^th^ grade), and 49 (14.5%) were Higher Secondary (12 ^th^ grade) and above. Occupation revealed the following participants: 14.8% were unemployed/homemakers/others, 79.6% were employed, and 5.6% were students. Regarding religion, 93.5% were Muslims, and 6.5% were Hindu. Among the respondents, 57.7% were married, 34.4% were unmarried, and 7.4% were divorced or separated. The majority (76.3%) were from nuclear families, and 23.7% were from families with multiple members (spouses/parents). Most of the respondents (68.6%) came from a family consisting of two to five family members, followed by 24.6% of single respondents, and 6.8% were from more than six family members. The subjects' socioeconomic status showed that 71.3% of respondents' earnings were below 10000 TK per month based on their monthly income. It also represents the results of the association between different categories of depression and various sociodemographic variables, where the significant association of depression was detected with age (p=0.013), religion (p=0.038), marital status (p<0.002), number of family members (p=0.040), and monthly income (p<0.001). Nevertheless, the variables like education, gender, occupation, and family type did not exhibit any association with depression among HIV-positive respondents.

**Table 1.** Demographic & socioeconomic features and Association between sociodemographic & socioeconomic factors with depression among people living with HIV.

	n (%)	Depression level				Chi-square	p-value
		None	Mild	Moderate	Severe		
**Age**							
18 to 30 years	121 (35.8%)	4 (3.3%)	4 (3.3%)	27 (22.3%)	86 (71.1%)	20.98	**0.013**
31 to 40 years	121 (35.8%)	0 (0.0%)	9 (7.4%)	47 (38.8%)	65 (53.7%)		
41 to 50 years	69 (20.4%)	0 (0.0%)	4 (5.8%)	23 (33.3%)	42 (60.9%)		
Above 50 years	27 (8.0%)	2 (7.4%)	2 (7.4%)	6 (22.2%)	17 (63.0%)		
**Sex**							
Male	297 (87.9%)	5 (1.7%)	16 (5.4%)	98 (33.0%)	178 (59.9%)	9.82	0.132
Female	20 (5.9%)	1 (5.0%)	2 (10.0%)	3 (15.0%)	14 (70.0%)		
**Transgender**	21 (6.2%)	0 (0.0%)	1 (4.8%)	2 (9.5%)	18 (85.7%)		
Level of education							
Illiterate	116 (34.3%)	3 (2.6%)	12 (10.3%)	35 (30.2%)	66 (56.9%)	10.35	0.111
Up to Secondary	173 (51.2%)	3 (1.7%)	7 (4.0%)	52 (30.1%)	111 (64.2%)		
Higher Secondary and above	49 (14.5%)	0 (0.0%)	0 (0.0%)	16 (32.7%)	33 (67.3%)		
**Occupation**							
Unemployed	50 (14.8%)	1 (2.0%)	2 (4.0%)	13 (26.0%)	34 (68.0%)	3.28	0.772
Employed	269 (79.6%)	5 (1.9%)	17 (6.3%)	85 (31.6%)	162 (60.2%)		
Student	19 (5.6%)	0 (0.0%)	0 (0.0%)	5 (26.3%)	14 (73.7%)		
**Religion**							
Muslim	316 (93.5%)	6 (1.9%)	19 (6.0%)	101 (32.0%)	190 (60.1%)	8.40	**0.038**
Hindu	22 (6.5%)	0 (0.0%)	0 (0.0%)	2 (9.1%)	20 (90.9%)		
**Marital status**							
Married	195 (57.7%)	5 (2.6%)	16 (8.2%)	70 (35.9%)	104 (53.3%)	20.66	**0.002**
Unmarried	118 (34.9%)	0 (0.0%)	3 (2.5%)	25 (21.2%)	90 (76.3%)		
Divorce or separated	25 (7.4%)	1 (4.0%)	0 (0.0%)	8 (32.0%)	16 (64.0%)		
**Types of family**							
Nuclear	258 (76.3%)	5 (1.9%)	12 (4.7%)	78 (30.2%)	163 (63.2%)	2.20	0.532
Joint	80 (23.7%)	1 (1.2%)	7 (8.8%)	25 (31.2%)	47 (58.8%)		
**Family size**							
Single	83 (24.6%)	0 (0.0%)	2 (2.4%)	18 (21.7%)	63 (75.9%)	13.21	**0.040**
2 to 5 members	232 (68.6%)	6 (2.6%)	14 (6.0%)	79 (34.1%)	133 (57.3%)		
6 and above	23 (6.8%)	0 (0.0%)	3 (13.0%)	6 (26.1%)	14 (60.9%)		
**Monthly income**							
<10,000 BDT	241 (71.3%	5 (83.3%)	12 (63.2%)	66 (64.1%)	158 (75.2%)	28.89	**<0.001**
10,001 – 20,000 BDT	94 (27.8%)	0 (0.0%)	6 (31.6%)	37 (35.9%)	51 (24.3%)		
>20,001 BDT	3 (0.9%)	1 (16.7%)	1 (5.3%)	0 (0.0%)	1 (0.5%)		

**Figure 1.**
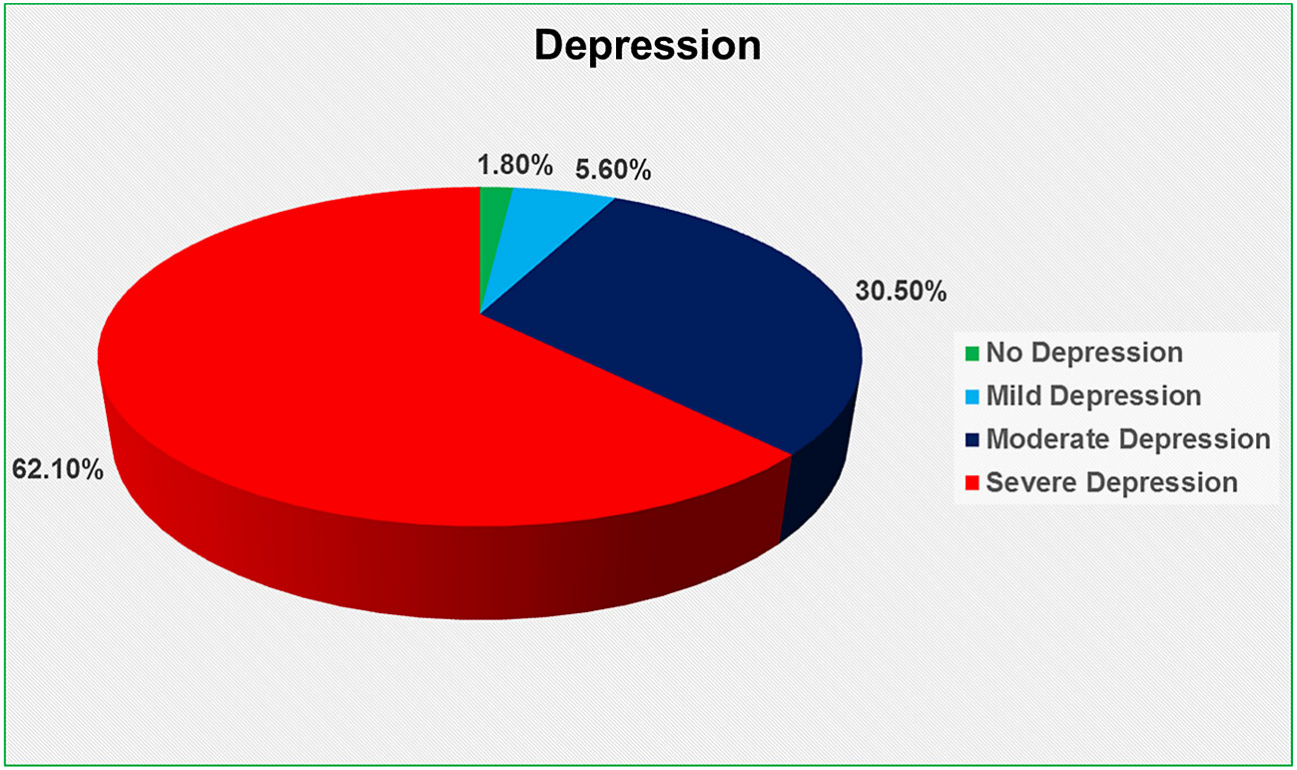
Depression level of the HIV-positive respondents.

The Beck Depression Inventory (BDI) scale was used to determine depression, which was divided into four categories: no depression (0–9), mild depression (10–16), moderate depression (17–29), and severe depression (30–63) ( Unnikrishnan, Jagannath, Ramapuram, Achappa, & Madi, 2012). We discovered that 62.1 percent had severe depression, 30.5 percent had moderate depression, 5.6 percent had mild depression, and only 1.8 percent had no depression ( Figure 1).

An adjusted multivariable model was created by forwarding stepwise logistic regression using the significant factors with the bivariate model. In our study, in the case of religion, Hindus were 4.9 times more prone to develop severe depression than their counterpart, Muslims (AOR=4.93; 95%CI: 1.09-22.24). Unmarried individuals had 1.9 times more chances to develop severe depression than married individuals (AOR=1.95; 95%CI: 1.00-3.80). Transgender people were more prone to develop severe depression than male and female respondents, but the association was not statistically significant in multivariable analysis. Similarly, students were more likely to develop severe depression than other occupations but did not find significant associations. Other variables such as education, family types, number of family members, and income were not significantly associated with depression in HIV patients ( Table 2).

**Table 2.** Unadjusted and adjusted analysis of factors associated with severe depression among the participants.

Variables	Crude			Adjusted ^1^		
	OR	95% CI	*p*-value	OR	95% CI	*p*-value
**Age**						
18-30 years	Ref.			Ref.		
31-40 years	0.47	0.27 – 0.80	0.006 *	0.71	0.37 – 1.36	0.306
41-50 years	0.63	0.34 – 1.18	0.150	0.98	0.47 – 2.03	0.963
Above 50 years	0.69	0.28 – 1.65	0.409	1.26	0.48 – 3.29	0.627
**Sex**						
Male	Ref.			Ref.		
Female	1.56	0.58 – 4.17	0.376	1.64	0.57 – 4.73	0.354
Transgender	4.01	1.15 – 13.91	0.029 *	1.59	0.38 – 6.60	0.518
**Level of education**						
Illiterate	Ref.			Ref.		
Up to Secondary	1.35	0.83 – 2.19	0.215	1.16	0.69 – 1.96	0.553
Higher Secondary and above	1.56	0.77 – 3.15	0.212	1.14	0.66 – 3.09	0.355
**Occupation**						
Unemployed	Ref.			Ref.		
Employed	0.71	0.37 – 1.35	0.301	0.95	0.46 – 1.95	0.892
Student	1.31	0.40 – 4.29	0.647	1.17	0.31 – 4.30	0.810
**Religion**						
Muslim	Ref.			Ref.		
Hindu	6.63	1.52 – 28.86	0.012 *	4.93	1.09 – 22.24	0.038 *
**Marital status**						
Married	Ref.			Ref.		
Unmarried	2.81	1.69 – 4.67	< 0.001 *	1.95	1.00 – 3.80	0.049 *
Divorced or separated	0.65	0.65 – 3.69	0.316	1.16	0.41 – 3.28	0.776
**Types of family**						
Nuclear	Ref.			Ref.		
Joint	0.83	0.49 – 1.38	0.476	0.77	0.42 – 1.43	0.420
**Family size**						
Single	Ref.			Ref.		
2 to 5 members	0.42	0.24 – 0.75	0.003 *	0.69	0.33 – 1.45	0.331
6 and above	0.49	0.18 – 1.31	0.157	0.75	0.25 – 2.28	0.620
**Monthly income**						
<10,000	Ref.			Ref.		
10,001-20,000	0.62	0.38 – 1.01	0.056	0.65	0.38 – 1.14	0.120
>20,001	0.26	0.02 – 2.93	0.278	0.17	0.01 – 2.48	0.198

^1^ Adjusted with age, sex, religion, marital status, and family members.

* Significant *p*-value at *p*<0.05.

## Discussion

The purpose of this study was to assess depression in PLWHA. Depression is associated with a wide ranges of chronic diseases ( Lotfaliany *et al.*, 2018; Louvardi *et al.*, 2020). However, in this study included 338 HIV-positive respondents, ranging from 18 to more than 50 years old, with a mean age of 35.6 years. The average age of participants in a similar study conducted in Sub-Saharan Africa was 38.9 years, slightly higher than ours ( van Coppenhagen & Duvenage, 2019). Chikezie *et al.* in Nigeria found that the average age of participants was 35.57 years, similar to our study ( Chikezie, Otakpor, Kuteyi, & James, 2013).

The Beck Depression Inventory was used in this study, and it was used to categorize depression into four categories: no depression, mild depression, moderate depression, and severe depression. We discovered that 62.1 percent of people had severe depression, 30.5 percent had moderate depression, 5.6 percent had mild depression, and 1.8 percent had no depression. A similar study conducted in Brazil found that the prevalence of no depression was 46.3 percent, mild depression was 17.7 percent, moderate depression was 22.7 percent. Severe depression was 13.3 percent, significantly lower than our study in moderate and severe depression levels ( Dal-Bó *et al.,* 2015). Another study in China found that 71.9 percent of people suffer from mild to severe depression ( Su *et al.,* 2013). In the north, west, and south of Iran, depression was found in 45 percent, 30 percent, and 56 percent of HIV patients, respectively. Furthermore, depression was prevalent in 25 percent of addicts and 58 percent of non-addicts, respectively ( Doosti-Irani, Moameri, Ahmadi-Gharaei, & Holakouie-Naieni, 2017).

Some differences in depression prevalence could be due to those countries' socio-cultural and economic contexts, such as income, political and social stability, strong familial support, and healthy social environments. This cross-sectional study found that males were suffering more from depression than females. The possible reasons could be that men are more likely to smoke, drink alcohol, eat unhealthily, and are often less aware of medical conditions and confront unemployment, economic hardship, etc. (Alkazemi, 2019). A study conducted in Kalafong Provincial Tertiary Hospital slightly differs from ours, where they found that females were more depressive than males (55.70% vs. 50.66%) ( van Coppenhagen & Duvenage, 2019). In addition, several studies also reported that women had more depression, anxiety, and stress, such as Gordillo *et al.* ( Gordillo *et al.*, 2009) Wisniewski *et al.* ( Wisniewski *et al.*, 2005) Rapaport *et al.* ( Rapaport, Clary, Fayyad, & Endicott, 2005) and Othman *et al.* ( Othman, Fadzil, Zakaria, Jaapar, & Husain, 2015).

This study revealed that participants whose monthly household income was less were at higher risk for depression; similar findings were reported by a study conducted at three hospitals in Ethiopia, which found that income less than 200 birr's was associated with depression ( Gupta *et al.*, 2010). This could be because people in low-income countries are pressured to rely on academics due to poverty-related factors, leading to increased domestic work and a lack of access to health education and awareness ( Al Jarad *et al.*, 2018). Deshmukh *et al.* conducted a study that backs up this claim ( Deshmukh *et al.*, 2017).

Dorsisa *et al.* (2020) found that married people are more depressed than unmarried people in Ethiopia ( Dorsisa, Ahimed, Anand, & Bekela, 2020), but we found that unmarried people are more likely to develop depression in our current study. Loneliness and a lack of mental support from partners to share the pain could be the cause, resulting in various negative thoughts. Our research found a link between age and depressive symptoms in people aged 18 to 30, and Abebe *et al.* (2019) found a similar link. Understanding and conceptualizing that their HIV status increases with age and transitioning to adulthood may be fraught with developmental challenges ( Abebe, Shumet, Nassir, Agidew, & Abebaw, 2019). In some studies, specific characteristics, such as age, employment status, and income level, have been linked to depression in PLWHA ( Nanni, Caruso, Mitchell, Meggiolaro, & Grassi, 2014; Rabkin, 2008; Eller *et al.,* 2014; Do *et al.,* 2014).

### Policy, practice and further research

Health promotion campaigns should incorporate a shift from fear to care, as this is important to treat PLWHA having depressive symptoms. As stigma and discrimination continue to be crucial factor that impedes prevention programs, policymakers need to strengthen the HIV/AIDS intervention and health education program in local communities in Bangladesh. Educating the population regarding the importance of mental health can play a significant role in responding to this menace. Education, knowledge, and awareness are believed to be the vanguard for this condition. Behavioral change strategies can be fruitful too. Future studies should also focus more on HIV/AIDS education or intervention programs that aim to increase the knowledge and awareness of the population in the communities, especially among rural communities.

## Conclusions

The current study found a high prevalence of depressive symptoms among HIV-positive patients in Bangladesh. In order to improve patient care and clinical outcomes, routine screening is critical in addressing this common psychiatric condition among HIV-positive populations. The Ministry of Health should develop guidelines to screen and treat depression among HIV patients. Because depression is so common among HIV-positive people, policymakers should include mental health programs in routine HIV care so that depression can be detected and treated early.

## Data availability statement

### Underlying data

Zenodo: HIV/AIDS-Depressive symptoms dataset; https://doi.org/10.5281/zenodo.5808314 ( Rabeya, 2021)

This project contains the following underlying data:

• Data.xls (raw data from questionnaires)

### Extended data

Zenodo: HIV/AIDS-Depression questionnaire. https://doi.org/10.5281/zenodo.5904418 ( Rabeya, 2022)

This project contains the following extended data:

• **Data file 1.** Copy of the survey administered to participants (in English).

Data are available under the terms of the Creative Commons Attribution 4.0 International license (CC-BY 4.0).

## Author contributions

All of the authors greatly aided the manuscript's development.
