# Supplementary material for: Depressive symptoms and their sociodemographic determinants among people living with HIV/AIDS in Bangladesh: a cross-sectional study
Source: F1000Res. 2023 Apr 17;11:239. Originally published 2022 Feb 25. [Version 3] doi: 10.12688/f1000research.108557.3 (PMC10186061; doi:10.12688/f1000research.108557.3)
Supplement: Peer review history

# Peer review: Reviewer response for version 3

**DOI:** 10.5256/f1000research.146499.r169892

**Published:** 2023-05-15

**Authors:** Collins Adu

**Affiliations:** 1 Center for Social Research in Health, University of New South Wales, Sydney, New South Wales, Australia; 2 College of Public Health, Medical and Veterinary Sciences, James Cook University, Townsville, Qld, Australia

Thank you very much. All my comments have been addressed to my satisfaction

Is the work clearly and accurately presented and does it cite the current literature?

Yes

If applicable, is the statistical analysis and its interpretation appropriate?

Yes

Are all the source data underlying the results available to ensure full reproducibility?

Yes

Is the study design appropriate and is the work technically sound?

Yes

Are the conclusions drawn adequately supported by the results?

Yes

Are sufficient details of methods and analysis provided to allow replication by others?

Partly

Reviewer Expertise:

Infectious diseases (HIV/AIDS); policy and prevention of violence against women; sexual violence; sexual and reproductive health

I confirm that I have read this submission and believe that I have an appropriate level of expertise to confirm that it is of an acceptable scientific standard.

# Peer review: Reviewer response for version 2

**DOI:** 10.5256/f1000research.138010.r154144

**Published:** 2023-01-16

**Authors:** Collins Adu

**Affiliations:** 1 Center for Social Research in Health, University of New South Wales, Sydney, New South Wales, Australia; 2 College of Public Health, Medical and Veterinary Sciences, James Cook University, Townsville, Qld, Australia

**Abstract**

- Under the background section. Please revise that section to inform the need for the study.
- Results: Can being a man be changed to male please? Male and Female are the accepted definition of terms for gender.

**Introduction**

- First paragraph: Sharp and Hahn (2011) recognized what?
- Second paragraph: Please provide  a reference for the definition of depression. Provide a reference to the statement depression is common among HIV-positive  people. Please, revise the last statement in second paragraph. It is too long to read with no clarification.
- Third paragraph: what is the full meaning of PLWHA? It was just introduced. Can you provide a recent literature on the last but one statement in paragraph 3. 1996 is too old.

**Methods**

- What research design was employed? Mixed-method or quantitative or qualitative.
- Please remove the last paragraph under the methods section and add it up to the ethics approval and consent to participate. You have to revise that section to avoid duplication of ideas.

**Discussion**

- Did you assess depression among people living with HIV? Was that the objective of the study?
- I suggest that the authors provide another section to write on the implication for policy, practice, and further research.
- The conclusion reads well but with the introduction of the implication for policy, practice, and further research section, it must be revised very well and be concise.

Is the work clearly and accurately presented and does it cite the current literature?

Yes

If applicable, is the statistical analysis and its interpretation appropriate?

Yes

Are all the source data underlying the results available to ensure full reproducibility?

Yes

Is the study design appropriate and is the work technically sound?

Yes

Are the conclusions drawn adequately supported by the results?

Yes

Are sufficient details of methods and analysis provided to allow replication by others?

Partly

Reviewer Expertise:

Infectious diseases (HIV/AIDS); policy and prevention of violence against women; sexual violence; sexual and reproductive health

I confirm that I have read this submission and believe that I have an appropriate level of expertise to confirm that it is of an acceptable scientific standard, however I have significant reservations, as outlined above.

# Response

**Published:** 2023-04-03

**Authors:** Md. Kamrul Hasan

**Affiliations:** North South University, Bangladesh

Comment-1:

 Under the background section. Please revise that section to inform the need for the study.

 Authors' response: Thank you. The authors revised accordingly. 

 Comment-2: 

 Results:

 Q. Can being a man be changed to male please? Male and Female are the accepted definition of terms for gender.

 Authors' response: We changed man to male in the revised version. Thank you. 

 Comment-3: 

 Introduction

 First paragraph:

 Q. Sharp and Hahn (2011) recognized what?

 Authors' response: Thank you. In the revised manuscript authors addressed and fixed this. 

 Comment-4:

 Second paragraph:

 Q. Please provide a reference for the definition of depression.

 Similar to (World Health Organization, 2018) definition

 Authors' response: Reference was provided. Thank you.

 Q. Provide a reference to the statement depression is common among HIV-positive people.

 (Deshmukh, Borkar, & Deshmukh, 2017). https://10.4103/2249-4863.222016

 Authors' response: Added. 

 Q. Please, revise the last statement in second paragraph. It is too long to read with no clarification.

 Psychiatric morbidity is more common in female with poor health who receive poor quality health care and have lacking emotional support from their family and friends.

 Authors' response: Revised. 

 Comment-5:

 Third paragraph:

 Q. what is the full meaning of PLWHA? It was just introduced. Can you provide a recent literature on the last but one statement in paragraph 3. 1996 is too old.

 https://doi.org/10.1371/journal.pone.0143749

 Authors' response: Addressed and fixed. 

 Comment-6:

 Methods

 Q. What research design was employed? Mixed-method or quantitative or qualitative. (observational study)

 Authors' response: It is a cross-sectional study. Thank you.

 Q. Please remove the last paragraph under the methods section and add it up to the ethics approval and consent to participate. You have to revise that section to avoid duplication of ideas.

 Authors' response: Addressed and fixed. 

 Comment-7:

 Discussion

 Q. Did you assess depression among people living with HIV? Was that the objective of the study?

 Author's response: Yes, the authors assessed depression among people living with HIV.

 Q. I suggest the authors provide another section to write on the implication for policy, practice, and further research.

 Author's response: Thank you. 

 The conclusion reads well but with the introduction of the implication for policy, practice, and further research section, it must be revised very well and be concise.

 Author's response: Revised.

# Peer review: Reviewer response for version 2

**DOI:** 10.5256/f1000research.138010.r149883

**Published:** 2022-09-12

**Authors:** Epari Venkatarao

**Affiliations:** 1 Department of Community Medicine, Institute of Medical & Sum Hospital, Siksha ‘O’ Anusandhan, Bhubaneswar, Odisha, India

Thanks.

 The authors have complied with the comments and have updated the changes in the article satisfactorily. 

 I have no further comments. 

 The article may be indexed.

 Thanks again.

Is the work clearly and accurately presented and does it cite the current literature?

Yes

If applicable, is the statistical analysis and its interpretation appropriate?

Yes

Are all the source data underlying the results available to ensure full reproducibility?

Yes

Is the study design appropriate and is the work technically sound?

Yes

Are the conclusions drawn adequately supported by the results?

Yes

Are sufficient details of methods and analysis provided to allow replication by others?

Yes

Reviewer Expertise:

Depression, Reproductive health, Clinical trials, NCDs, Physical activity promotion, Oncology. etc.

I confirm that I have read this submission and believe that I have an appropriate level of expertise to confirm that it is of an acceptable scientific standard.

# Peer review: Reviewer response for version 1

**DOI:** 10.5256/f1000research.119952.r145206

**Published:** 2022-08-09

**Authors:** Epari Venkatarao

**Affiliations:** 1 Department of Community Medicine, Institute of Medical & Sum Hospital, Siksha ‘O’ Anusandhan, Bhubaneswar, Odisha, India

This is an important study documenting the burden of depressive symptoms among PLHIV, which can contribute to policy formulation, both locally and globally. The study has been conducted carefully and technically the study design is appropriate to respond to the study objectives. A review of the literature has been done optimally.

 However, I have just two comments

 Table 1 was not needed, it can be merged with table 2 as a composite table and that would reduce the article space in the journal.

 Secondly, under the discussion section one or two articles relating to depression among people with other chronic diseases, might add value to the article.

Is the work clearly and accurately presented and does it cite the current literature?

Yes

If applicable, is the statistical analysis and its interpretation appropriate?

Yes

Are all the source data underlying the results available to ensure full reproducibility?

Yes

Is the study design appropriate and is the work technically sound?

Yes

Are the conclusions drawn adequately supported by the results?

Yes

Are sufficient details of methods and analysis provided to allow replication by others?

Yes

Reviewer Expertise:

Depression, Reproductive health, Clinical trials, NCDs, Physical activity promotion, Oncology. etc.

I confirm that I have read this submission and believe that I have an appropriate level of expertise to confirm that it is of an acceptable scientific standard, however I have significant reservations, as outlined above.

# Response

**Published:** 2022-08-31

**Authors:** Md. Kamrul Hasan

**Affiliations:** North South University, Bangladesh

**Comment-1**: Table 1 was not needed, it can be merged with table 2 as a composite table and that would reduce the article space in the journal.

**Author's Response**: Thanks for the suggestion. As suggested the authors merged table 1 and table 2 and add a composite table in the revised (new version) manuscript. 

**Comment-2**: Secondly, under the discussion section one or two articles relating to depression among people with other chronic diseases, might add value to the article.

**Author's Response**: Thanks. The authors added information relating to depression among people with other chronic diseases with appropriate citations in the revised (new version) manuscript.
